# Epidemiological, molecular, and evolutionary characteristics of G1P[8] rotavirus in China on the eve of RotaTeq application

**DOI:** 10.3389/fcimb.2024.1453862

**Published:** 2024-12-09

**Authors:** Rui Peng, Mengxuan Wang, Saleha Shahar, Guangping Xiong, Qing Zhang, Lili Pang, Hong Wang, Xiangyu Kong, Dandi Li, Zhaojun Duan

**Affiliations:** ^1^ National Key Laboratory of Intelligent Tracking and Forecasting for Infectious Diseases (NITFID), National Health Commission Key Laboratory for Medical Virology and Viral Diseases, National Institute for Viral Disease Control and Prevention, Chinese Center for Disease Control and Prevention, Beijing, China; ^2^ Department of Biosciences, Faculty of Sciences, Universiti Teknologi Malaysia, Johor Bahru, Malaysia

**Keywords:** rotavirus, whole genome, epidemiology, evolution, selection pressure, neutralizing antigenic epitopes

## Abstract

**Introduction:**

This study, conducted in China prior to RotaTeq’s launch, examined the epidemiological, molecular, and evolutionary features of the G1P[8] genotype RVA in children admitted with diarrhea, to aid in evaluating its efficacy and impact on G1P[8] RVA in China.

**Methods:**

Data from the Chinese viral diarrhea surveillance network were collected from January 2016 to December 2018. RVA strains identified as the G1P[8] genotype were subjected to whole-genome sequencing. Neutralizing epitope, amino acid selection pressure, and evolution dynamics analyses on VP7 and VP4 were performed using BioEdit v.7.0.9.0 and PyMOL v.2.5.2, four algorithms (MEME, SLAC, FEL, and FUBAR) in the Datamonkey online software, and the MCMC model in BEAST v. 1.10.4, respectively. Phylogenetic and identity features of 11 genes were assessed by DNAStar and MEGA v.7.

**Results:**

Results showed that the detection rate of G1P[8] in China from 2016 to 2018 was generally low with significant seasonality. The whole genome of G1P[8] of four 2016 childhood diarrhea specimens was successfully sequenced. Phylogenetic and neutralizing epitope analysis showed that Rotavin-M1 might have better protection on G1P[8] prevalent in China than Rotarix and RotaTeq. Two conserved N-glycosylation sites on VP7 of Chinese G1P[8] might affect the protective effect of the vaccine. Evolution rate and selection pressure analysis identified the possibility of rapidly evolving and adapting to the new environment introduced by vaccines of G1P[8], whereas positive selection specific to VP4 indicated the potential tendency to select for dominant traits. Identity and phylogeny analysis showed that Chinese G1P[8] from before 2018 was generally stable with possible genetic recombination among local strains.

**Discussion:**

These findings not only are of great significance for predicting the prevalence of G1P [8] in China, but also provide data reference for evaluating rotavirus vaccine efficacy.

## Introduction

Rotaviruses are icosahedral, triple-coated viruses belonging to the family *Reoviridae* (Uprety et al., 2021). Group A rotavirus (RVA) is the leading cause of severe diarrhea in children worldwide, accounting for 19.11% of deaths from diarrhea in 2019 (Du et al., 2022). It caused more than 258,000,000 [95% uncertainty interval (95% UI): 193,000,000–341,000,000] infections and 128,500 deaths (95% UI 104,500–155,600) in children under 5 years of age worldwide in 2016 (Troeger et al., 2018; Du et al., 2022). The rotaviruses have an 11-segmented double-stranded RNA genome that encodes six structural proteins (VP1–VP4, VP6, and VP7) and six nonstructural proteins (NSP1–NSP6) (Matthijnssens et al., 2008). RVA is classified into G and P genotypes based on the VP7 and VP4 genes encoding two coat proteins (Matthijnssens et al., 2008). To date, 42 G and 58 P genotypes have been classified worldwide by the Rotavirus Classification Working Group (RCWG) (https://rega.kuleuven.be/cev/viralmetagenomics/virus-classification/rcwg) (Malakalinga et al., 2019; Lestari et al., 2020).

Epidemiological studies have shown that the most frequently detected human RVA genotypes worldwide are G1P[8], G2P[4], G3P[8], G4P[8], G9P[8], and G12P[8] (Antoni et al., 2023). Globally, the most frequent genotypes identified after weighting were G1P[8] (31%), G1P[6] (8%), and G3P[8] (8%) (Antoni et al., 2023). G1P[8] is the most common and prevalent genotype and remains the most dominant in many regions of the world (Heylen et al., 2014; Jain et al., 2016; Zeller et al., 2017; Kaplon et al., 2018; Tian et al., 2018; Antoni et al., 2023). In China, G1P[8] was the dominant strain from 1983 to 2000, although from 2000 to 2007, G3P[8] RVA rapidly increased as the dominant strain and gradually replaced G1P[8] (Tian et al., 2018). Rotavirus continues to evolve, and changes in the VP7 and VP4 proteins’ neutralizing epitopes may eventually cause antibody escape mutants to arise (Kirkwood, 2010; Bucardo et al., 2012; Zeller et al., 2012; Jere et al., 2018; Ogden et al., 2019). Prior research has noted an apparent recurrence of a 10-year cycle in the rotavirus genotype circulation, with Wa-like and DS-1-like genotypes switching over time (Carvalho-Costa et al., 2019a). In summary, to effectively prevent the prevalence of RVA, we need to conduct continuous monitoring of its genotypes to gain a deeper understanding of their changing patterns.

The attenuated Lanzhou lamb rotavirus vaccine (LLR, Lanzhou Institute of Biological Products, China, G10/P[15]) has been introduced into the immunization program in China to prevent RVA infection in children since 2001 (Chinese Preventive Medicine Association, 2021). Yet, the vaccine protection efficacy was low: at least one dose of the LLR vaccine is effective in 52% to 88% of patients with severe rotavirus gastroenteritis (RVGE) and 35% of patients with any degree of RVGE (Deen et al., 2018). In November 2018, the pentavalent human–bovine reassortant rotavirus vaccine (RotaTeq/RV5, Merck, Rahway, NJ, USA) was introduced in China as a complementary vaccine to prevent RVA, particularly for serotypes G1–G4 and P[8] (Burke et al., 2019; Chinese Preventive Medicine Association, 2021; Peng et al., 2023).

Studies showed that the use of two widely available rotavirus vaccines, including RotaTeq, had greatly reduced the incidence and mortality of RVA-induced diarrhea in children worldwide (Coria de la, 2006; Vesikari et al., 2006). On the other hand, long-term use of rotavirus vaccines was also associated with the re-emergence of disappeared RVA genotypes and the production of new or uncommon RVA genotypes (Dormitzer et al., 2004; Aliabadi et al., 2019; Hungerford et al., 2019; Donato et al., 2022). These highlights what could be the reciprocal influence or effect of rotavirus vaccines and their protective role on the prevalence of various RVA genotypes, which remains inadequately understood. This study sought to explore the epidemiological, molecular, and evolutionary features of G1P[8] RVA on the eve of RotaTeq vaccine entry into China. The insights gained from this research will be valuable for future work assessing the effectiveness of RotaTeq in China and its potential influence of the genotype.

## Materials and methods

### Epidemiological data

According to the National Viral Diarrhea Surveillance Program (2007 Revision) issued by the Chinese CDC, national surveillance sites were established in 20 provinces in China to carry out surveillance of viral diarrhea cases in children under 5 years of age. Stool samples were collected under aseptic condition from hospitalized children experiencing diarrhea for up to 3 days. A total of 29,388 specimens were tested from January 2016 to December 2018. The detection rate of RVA was calculated as the number of RVA ELISA positive samples / the number of specimens tested × 100%. Detection rate of G1P[8] was calculated as the number of G1P[8] specimens / the number of specimens tested × 100%. G1P[8] composition ratio was calculated as the number of positive G1P[8] / the number of RVA ELISA positive samples× 100%.

### ELISA and G/P genotyping of Rotavirus A

Stool suspension (10%) was prepared by mixing 0.1 mL or a pea-sized stool sample to 0.9 mL of phosphate buffer. The sample was homogenized by vortex for 3 min, centrifuged at 5,000×*g* for 10 min, and stored at −20°C until use. RVA antigen was detected from the homogenized stool samples using ProSpecT Rotavirus Microplate Assay (R240396, Thermo Fisher Scientific, USA) according to manufacturer instructions.

Subsequently, G1P[8] genotyping was done by semi-nested RT-PCR on RVA ELISA-positive samples. First, total nucleic acid was extracted from 200 μL of stool suspension using the Tianlong Nucleic Acid Automatic Extractor (NP968, Xi’an Tian Long Technology Co., Ltd) and the nucleic acid extraction kit CqEx-DNA/RNA Virus (CDC) and used for G/P genotyping as previously reported (Gouvea et al., 1990; Iturriza-gómara et al., 2004). Briefly, the 881 bp of VP7 gene (or 663 bp of VP4 gene) was amplified using specific primers VP7F and VP7R (or VP4 and VP4R) using reverse transcription-PCR (RT-PCR). Then, using the RT-PCR products as templates, PCR amplification was performed with specific primers for the conserved sequence of the G1 VP7 gene (or P[8] VP4 gene), resulting in a product of 618 bp (or 224 bp). Finally, the RVA G1P[8] genotype was confirmed based on the electrophoresis fragment size. Database was established with EpiData 3.0. Data organization was conducted using Excel 2010. Data analysis was carried out by SPSS 26.0. *p* < 0.05 was considered statistically significant.

### Whole-genome sequencing

Whole-genome sequencing was conducted on the RVA that was identified as the G1P[8] genotype in fecal samples. Briefly, the 11 RVA gene fragments were amplified separately by the QIAGEN OneStep RT-PCR Kit (HB-0454, QIAGEN) using primers specifically designed for each gene in the previous report (Mijatovic-Rustempasic et al., 2011). The PCR reaction conditions for VP1, VP2, VP3, VP4, and NSP5 were as follows: 50°C for 30 min, 95°C for 15 min; 35 cycles of 94°C for 30 s, 42°C for 30 s, 72°C for 1 min; 72°C for 7 min. The PCR reaction conditions for VP6, VP7, NSP1, NSP2, and NSP3 were as follows: 50°C for 30 min, 95°C for 15 min; 35 cycles of 94°C for 1 min, 42°C for 1 min, 72°C for 1 min 20 s; 72°C for 10 min. The RT-PCR products were electrophoresed using 1.5% agarose gel and observed by an ultraviolet transmission analyzer. Finally, the PCR products were sent to Tingke Biotechnology Co., Ltd. for sequencing.

### Analysis of neutralizing antigenic epitopes on deduced VP7 and VP4 amino acid sequences

The neutralizing antigenic epitopes on VP7/VP4 of G1P[8] amplified from the stool samples in this study and other Chinese G1P[8] available in GenBank (between 2004 and 2018) were compared and analyzed using MEGA v.7 and BioEdit v.7.0.9.0. The information about these sequences for neutralizing epitope analysis is listed in **Supplementary Table 1**. The VP7/VP4 neutralizing epitopes of Chinese G1P[8] were also compared with the homologous monovalent RVA vaccines, the Rotarix and Rotavin-M1, and the pentavalent RotaTeq vaccine. Finally, the three-dimensional structural differences between Chinese G1P[8] RVA and the homologous G1P[8] RVA vaccines were determined using protein database files 1KQR (Dormitzer et al., 2002) and 3FMG (Venkataram Prasad and Chiu, 1994) and PyMOL v.2.5.2.

### Selection pressure analysis of VP7 and VP4 proteins

Positive selection pressure sites and negative selection pressure sites were screened in 281 amino acids from site 10 aa to 290 aa of the VP7 and in 747 amino acids from position 23 aa to 769 aa of the VP4 of G1P[8] RVA in China before 2018 with four different algorithms (MEME, SLAC, FEL, and FUBAR) using the Datamonkey online software (Datamonkey Adaptive Evolution Server) (Kosakovsky Pond and Frost, 2005; Murrell et al., 2012). Positive selection pressure sites were determined with *p*-values <0.1 (MEME, SLAC, and FEL) or posterior probabilities >90% (FUBAR). The same result obtained by at least three algorithms was defined as integrated and considered credible. The information about these sequences for selection pressure analysis is listed in **Supplementary Table 1**.

### Bayesian evolutionary analysis of VP7 and VP4 genes

The evolutionary behavior of the G1-VP7 and P[8]-VP4 genes was analyzed on 167 VP7 and 130 VP4 gene sequences from different countries and time zones as reference sequences using the MCMC model in BEAST v. 1.10.4. A root-to-tip regression analysis using TempEst v1.5.3 revealed a positive correlation between genetic divergence and sampling time, indicating that the dataset is suitable for phylodynamic analysis with tip-dating calibration (Rambaut et al., 2016) (**Supplementary Table 2**). The most suitable models were determined through Bayes factor analysis using BEAST v1.10.4 and Tracer v1.6 (Newton and Raftery, 1994) (**Supplementary Tables 3**, **4**). Model comparisons were made based on marginal likelihood (with standard error estimated from bootstraps). Lastly, the evolution rate (nucleotide substitution/site/year), the most recent common ancestor (TMRCA), and Bayesian evolutionary dynamics of VP7 and VP4 were evaluated with nucleotide substitution model GTR+G, the Uncorrelated Relaxed Lognormal molecular clock model, and the Bayesian skyline coalescent tree prior model. In Tracer v.1.7.2, the MCMC was run 100,000,000 times to increase the effective sample size (ESS) to over 200, with sampling conducted every 1,000 steps. The maximum clade credibility (MCC) tree was annotated in TreeAnnotator v1.10.4 and displayed using FigTree v.1.4.3. Information regarding these sequences for Bayesian evolutionary analysis is listed in **Supplementary Table 1**.

### Whole-genome phylogenetic analysis

The phylogenetic and identity characteristics of G1P[8] before the introduction of RotaTeq into China were determined by analyzing whole-genome sequences of Chinese G1P[8] in this study from stool samples together with vaccines strains, other Chinese sequences before 2018, and other countries’ strains available in GenBank (**Supplementary Table 5**). Sequence splicing was performed with the Seqman software, genotyping was performed with the online tool RotaC V2.0, and identity analysis was performed with DNAStar. Maximum likelihood phylogenetic trees (ML tree) were established with MEGA v.7 using best-fit models T92 + G (NSP, NSP3, NSP5, VP4, and VP7), T92 + G + I (NSP2, VP1, VP3, and VP6), and TN93 + G (VP2) with 1,000 bootstrap replicates. The lineages were identified based on previous reports (Bonica et al., 2015; Santos et al., 2019; Fujii et al., 2020).

## Results

### Epidemiological characteristics of G1P[8] RVA infection

From 2016 to 2018, 4,309 of 15,374 stool specimens from children under 5 years of age hospitalized with diarrhea in China were positive for RVA, including 121 of the G1P[8] genotype. The detection rates of RVA and G1P[8] among children hospitalized with diarrhea were 28.03% (4,309/15,374) and 0.79% (121/15,374), respectively, whereas the composition ratio of G1P[8] among the RVA-positive diarrhea was 2.81% (121/4,309) (**Table 1**). By year, the composition ratio of the G1P[8] genotype in children with RVA diarrhea in China was 2.59% (36/1,388) for 2016, 3.89% (58/1,491) for 2017, and 1.89% (27/1,430) for 2018. In general, the G1P[8] genotype composition ratio fluctuated every year with 2018 being the lowest (**Table 1**).

**Table 1:** G1P [8] RVA infections from 2016 to 2018.

		Tested specimens	ELISA positive	G1P[8]	RVA detection rate (%)	G1P[8] detection rate (%)	G1P[8] composition ratio (%)
Year	2016	5,305	1,388	36	26.16	0.68	2.59
	2017	4,775	1,491	58	31.23	1.21	3.89
	2018	5,294	1,430	27	27.01	0.51	1.89
Total		15,374	4,309	121	28.03	0.79	2.81

**Figure 1:**
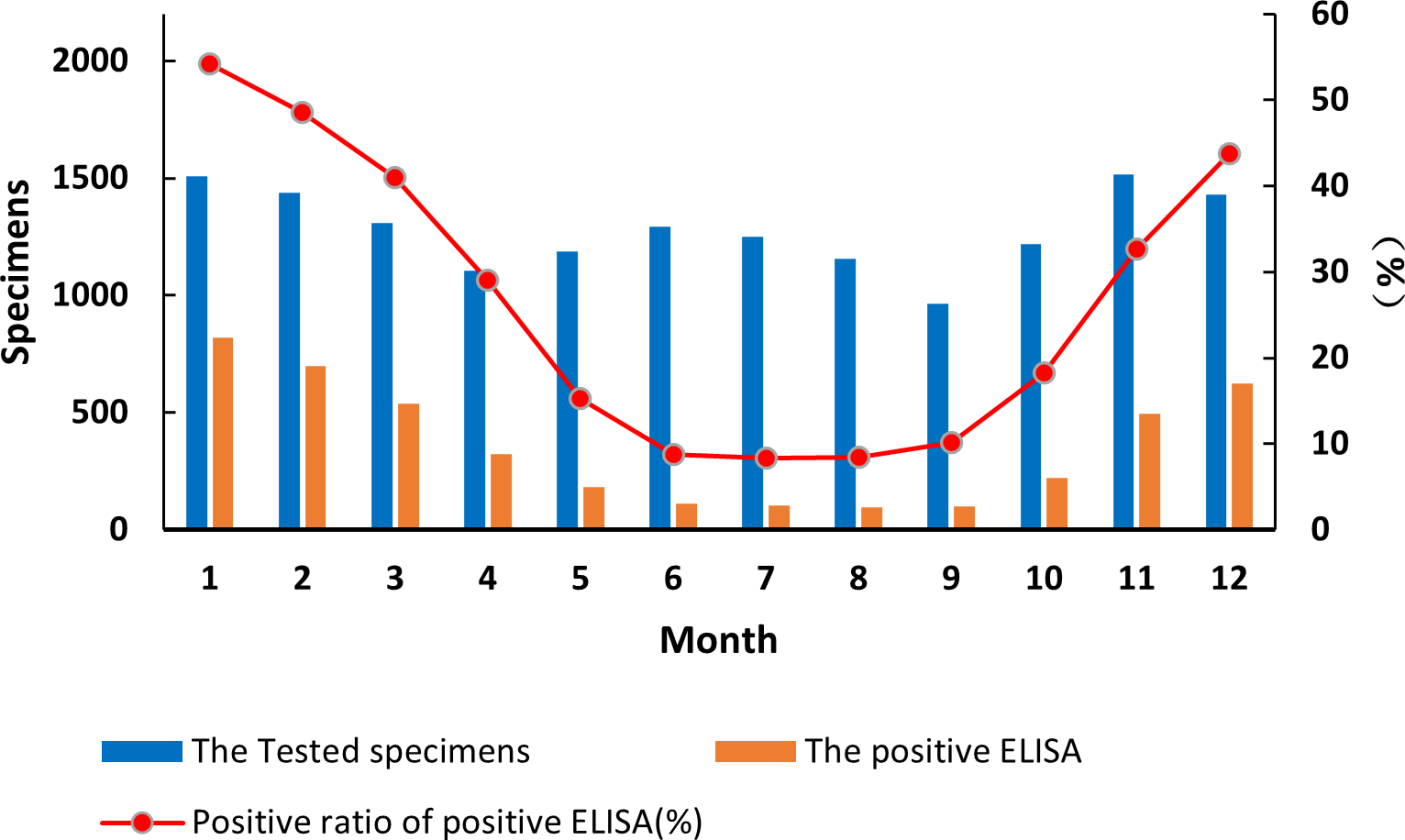
Incidence of rotavirus-positive stools from all acute gastroenteritis in under 5 years of age from 2016 to 2018. Specimen numbers show total samples for all 3 years each month.

By month, diarrhea from acute gastroenteritis cases in children under 5 years of age fluctuated in a biphasic pattern. Cases increased and decreased in two cycles with occurrences peaked on June–July and November–January (**Figure 1**). China is a large country with four climate zones (equatorial, tropical, subtropical, and moderate climates) shaping the country’s complex seasons. Across the vast landscape and climate zone, June–July is the peak of summer and December–February is winter. The detection rate of RVA in the diarrhea stools ranged from 8.33% to 54.24%. Interestingly, RVA incidences occurred in a single cycle with cases peaked in the winter (December–February) and cases at its lowest point in summer to autumn (June–September) (**Figure 1**). The differences among RVA detection rates by month were statistically significant (χ² = 2,120, *p* < 0.05). The monthly sample counts, RVA-positive cases, and G1P[8] RVA-positive cases from 2016 to 2018 are detailed in **Supplementary Table 6**. The results of the statistical analysis are presented in **Supplementary Table 7**.

**Figure 2:**
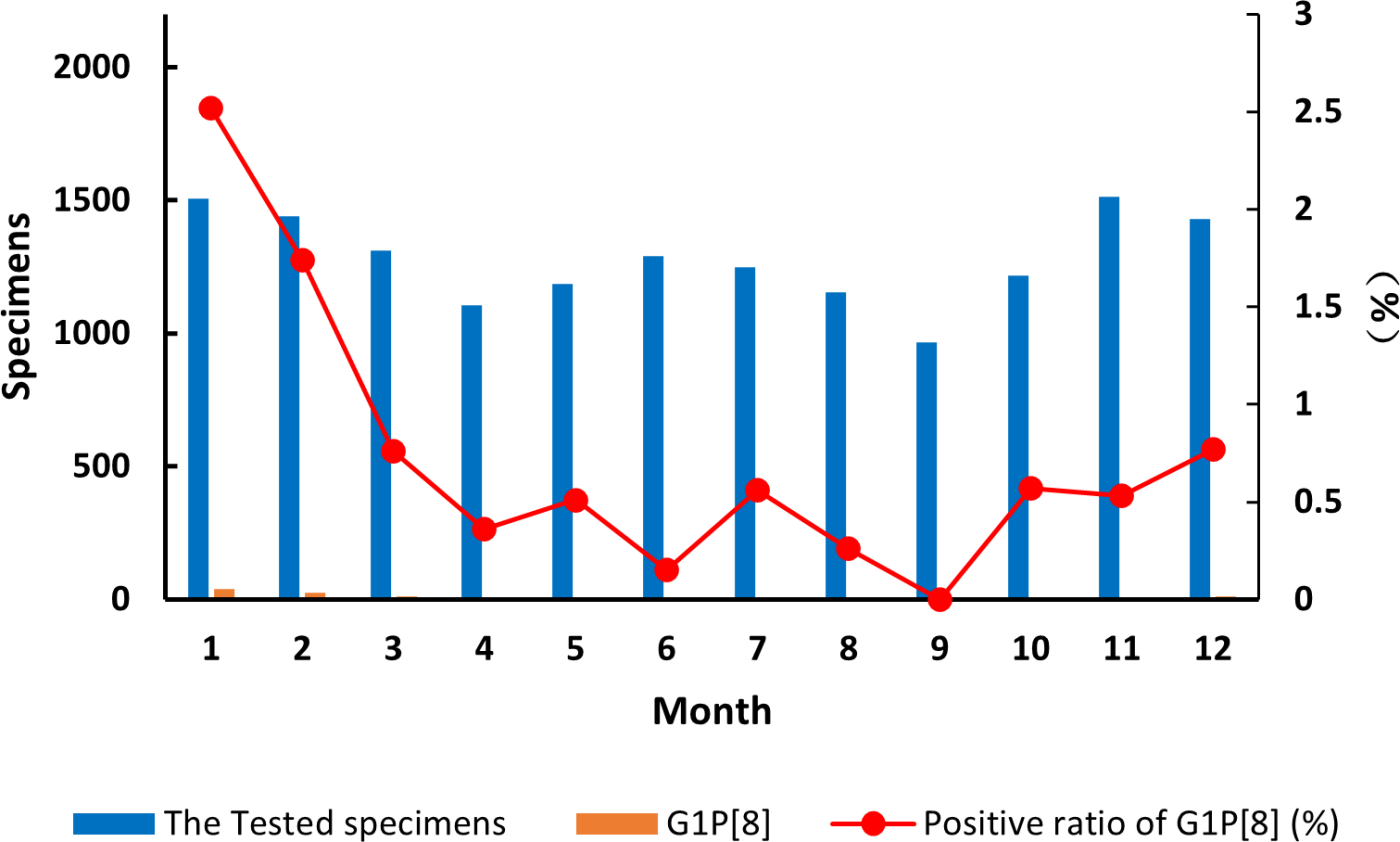
Incidence of the G1P[8] genotype (line graph) from all acute gastroenteritis in under 5 years of age from 2016 to 2018. Specimen numbers show total samples for all 3 years each month.

**Figure 3:**
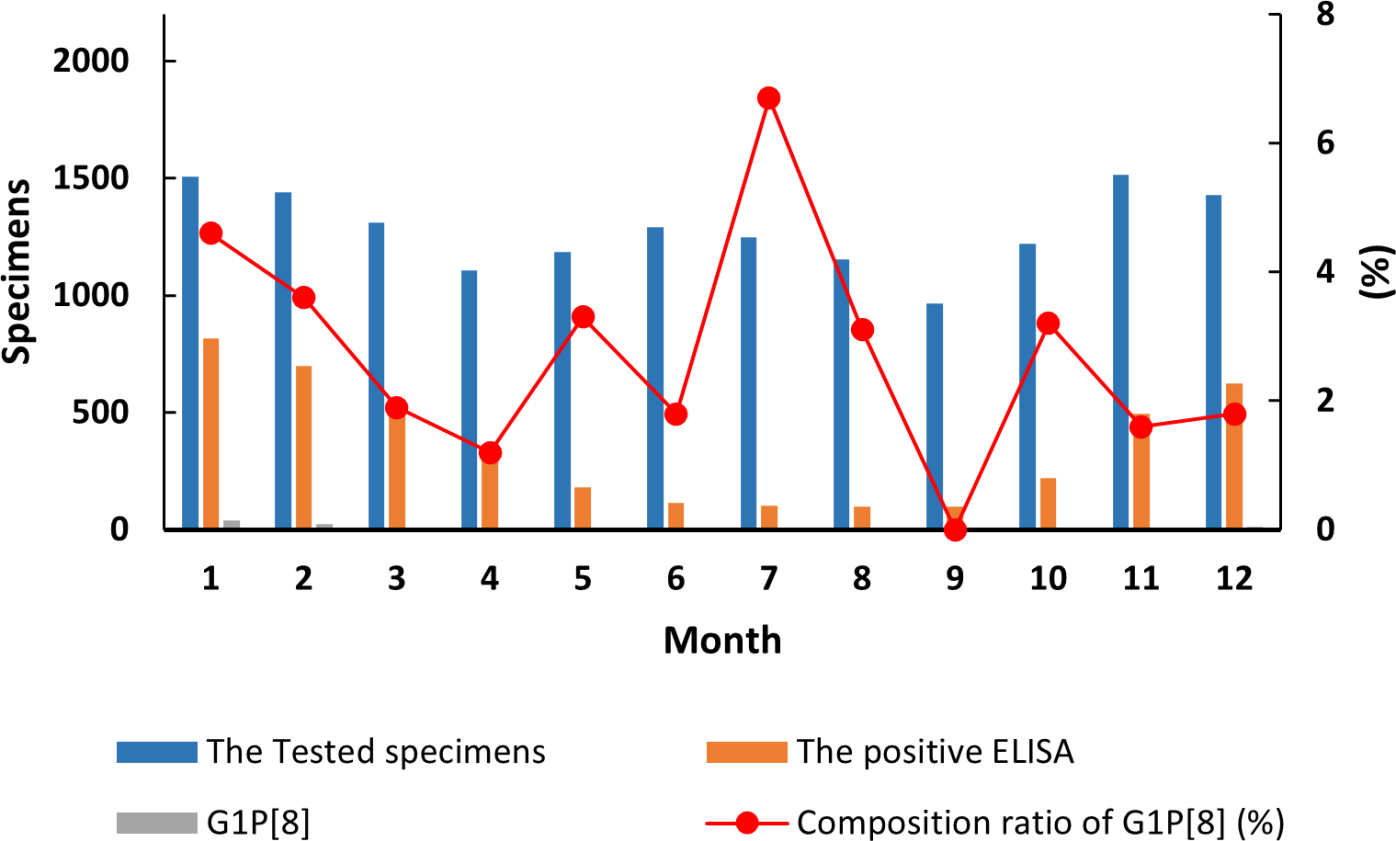
Incidence of the G1P[8] genotype (line graph) from all rotavirus-positive stools in under 5 years of age from 2016 to 2018. Specimen numbers show total samples for all 3 years each month.

The detection of the G1P[8] genotype from all acute gastroenteritis cases ranged from 0% to 2.52% and differed each month (χ² = 99.627, *p* < 0.05) (**Supplementary Table 7**). Detection rates fluctuated, showing a single cycle trend as was the RVA cases in general (**Figure 2**). However, when aggregated specifically by RVA cases, the G1P[8] genotype did not show a clear seasonal cycle and instead peaked erratically in January, May, July, and October (**Figure 3**). The differences among G1P[8] composition ratios by month were statistically significant (χ² =30.809, *p* < 0.05) (**Supplementary Table 7**).

### Genotyping

In this study, the whole genome of four G1P[8] strains from the stool samples collected in 2016 was successfully amplified and sequenced (reference number GS16622068, GS16622078, GS16622105, and GS16622118, subsequently referred to here as GS RVA). The nucleoside sequences were submitted to GenBank with accession numbers OQ472800–OQ472843. RotaC V.2.0 rotavirus genotyping online tool identified these GS RVA constellations as G1-P[8]-I1-R1-C1-M1-A1-N1-T1- E1-H1.

### Analysis of deduced neutralizing antigenic epitopes

Rotavirus VP7 contains 29 neutralizing antigenic epitopes, which are in three neutralizing antigenic epitope regions (7-1a, 7-1b, and 7-2) (Venkataram Prasad and Chiu, 1994). The VP7 neutralizing antigenic epitopes for GS RVA were conserved with other Chinese G1P[8] of earlier years showing only a few changes. Overall, the Chinese G1P[8] varied only by two amino acids at sites 91, 94, 96, 100, and 148. Compared to vaccine strains, the Chinese G1P[8] varied at 10 sites, 91, 94, 96, 97, 100, 123, 291, 147, 148, and 217. In detail, Rotarix varied from all the Chinese G1P[8] at S123N, K291R, and M217T and varied from some at sites 91, 94, 96, 100, and 148. These changes were the same when compared to RotaTeq with additional mutations at D97E and S147N. On the other hand, Rotavin-M1 varied from only some of the Chinese G1P[8] and at sites 91, 94, 96, 100, and 148 (**Table 2**). By this, the variation between Chinese strains and Rotavin-M1 were lesser (5 changes) compared to Rotarix and RotaTeq (8 and 10 changes). Additionally, two conserved N-glycosylation sites were found on Chinese G1P[8] at 69 aa and 238 aa close to the antigenic epitope or located at a possible neutralizing antibody escape site.

**Table 2:** Presumed VP7 neutralizing antigen epitope differences between Chinese G1P[8] RVA and G1P[8] vaccines.

Strains	7-1a														7-1b						7-2								
	87	91	94	96	97	98	99	100	104	123	125	129	130	291	201	211	212	213	238	242	143	145	146	147	148	190	217	221	264
RV1/G1 (Rotarix)	T	T	N	G	E	W	K	D	Q	S	V	V	D	K	Q	N	V	D	N	T	K	D	Q	N	L	S	M	N	G
RV5/G1 (RotaTeq)	T	T	N	G	D	W	K	D	Q	S	V	V	D	K	Q	N	V	D	N	T	K	D	Q	**S**	L	S	M	N	G
Rotavin-M1/G1	T	T	S	G	E	W	K	D	Q	N	V	V	D	R	Q	N	V	D	N	T	K	D	Q	N	L	S	T	N	G
GS16622068/2016	T	N	S	G	E	W	K	D	Q	N	V	V	D	R	Q	N	V	D	N	T	K	D	Q	N	L	S	T	N	G
GS16622078/2016	T	N	S	S	E	W	K	D	Q	N	V	V	D	R	Q	N	V	D	N	T	K	D	Q	N	L	S	T	N	G
GS16622105/2016	T	N	S	G	E	W	K	D	Q	N	V	V	D	R	Q	N	V	D	N	T	K	D	Q	N	L	S	T	N	G
GS16622118/2016	T	N	S	S	E	W	K	D	Q	N	V	V	D	R	Q	N	V	D	N	T	K	D	Q	N	L	S	T	N	G
Fuzhou18-194/2018	T	T	N	G	E	W	K	D	Q	N	V	V	D	R	Q	N	V	D	N	T	K	D	Q	N	F	S	T	N	G
Fuzhou18-262/2018	T	T	N	G	E	W	K	D	Q	N	V	V	D	R	Q	N	V	D	N	T	K	D	Q	N	F	S	T	N	G
Fuzhou18-195/2018	T	T	S	G	E	W	K	E	Q	N	V	V	D	R	Q	N	V	D	N	T	K	D	Q	N	L	S	T	N	G
Fuzhou18-216/2018	T	T	S	G	E	W	K	E	Q	N	V	V	D	R	Q	N	V	D	N	T	K	D	Q	N	L	S	T	N	G
Fuzhou18-242/2018	T	T	S	G	E	W	K	E	Q	N	V	V	D	R	Q	N	V	D	N	T	K	D	Q	N	L	S	T	N	G
SC18511002/2018	T	T	S	G	E	W	K	E	Q	N	V	V	D	R	Q	N	V	D	N	T	K	D	Q	N	L	S	T	N	G
SC18511004/2018	T	T	S	G	E	W	K	E	Q	N	V	V	D	R	Q	N	V	D	N	T	K	D	Q	N	L	S	T	N	G
SC18511045/2018	T	T	S	G	E	W	K	E	Q	N	V	V	D	R	Q	N	V	D	N	T	K	D	Q	N	L	S	T	N	G
SC18511073/2018	T	T	S	G	E	W	K	E	Q	N	V	V	D	R	Q	N	V	D	N	T	K	D	Q	N	L	S	T	N	G
JL18221009/2018	T	T	S	G	E	W	K	E	Q	N	V	V	D	R	Q	N	V	D	N	T	K	D	Q	N	L	S	T	N	G
E5365/2017	T	T	N	G	E	W	K	D	Q	N	V	V	D	R	Q	N	V	D	N	T	K	D	Q	N	F	S	T	N	G
F1530/2017	T	T	N	G	E	W	K	D	Q	N	V	V	D	R	Q	N	V	D	N	T	K	D	Q	N	F	S	T	N	G
F1199/2016	T	T	N	G	E	W	K	D	Q	N	V	V	D	R	Q	N	V	D	N	T	K	D	Q	N	F	S	T	N	G
Km15009/2015	T	T	S	G	E	W	K	E	Q	N	V	V	D	R	Q	N	V	D	N	T	K	D	Q	N	L	S	T	N	G
SC1/2014	T	T	N	G	E	W	K	D	Q	N	V	V	D	R	Q	N	V	D	N	T	K	D	Q	N	F	S	T	N	G
F398/2014	T	T	N	G	E	W	K	D	Q	N	V	V	D	R	Q	N	V	D	N	T	K	D	Q	N	F	S	T	N	G
WZ202/2013	T	N	S	G	E	W	K	D	Q	N	V	V	D	R	Q	N	V	D	N	T	K	D	Q	N	L	S	T	N	G
SC2/2013	T	N	S	G	E	W	K	D	Q	N	V	V	D	R	Q	N	V	D	N	T	K	D	Q	N	L	S	T	N	G
Q1135/2012	T	T	N	G	E	W	K	D	Q	N	V	V	D	R	Q	N	V	D	N	T	K	D	Q	N	F	S	T	N	G
Q348/2011	T	N	S	G	E	W	K	D	Q	N	V	V	D	R	Q	N	V	D	N	T	K	D	Q	N	L	S	T	N	G
Chi-87/2007	T	N	S	G	E	W	K	D	Q	N	V	V	D	R	Q	N	V	D	N	T	K	D	Q	N	L	S	T	N	G
Chi-77/2007	T	N	S	G	E	W	K	D	Q	N	V	V	D	R	Q	N	V	D	N	T	K	D	Q	N	L	S	T	N	G
R588/2005	T	N	S	G	E	W	K	D	Q	N	V	V	D	R	Q	N	V	D	N	T	K	D	Q	N	L	S	T	N	G
Y128/2004	T	N	S	G	E	W	K	D	Q	N	V	V	D	R	Q	N	V	D	N	T	K	D	Q	N	L	S	T	N	G
98SH53/1998	T	N	S	G	E	W	K	D	Q	N	V	V	D	R	Q	N	V	D	N	T	K	D	Q	N	L	S	T	N	G

Amino acid residues highlighted in blue were those different from all three vaccines Rotarix (RV1), RotaTeq (RV5), and Rotavin-M1. Amino acid residues different from both Rotarix and RotaTeq were marked with green. Amino acid residues only different from RotaTeq or Rotavin-M1 were marked with red and yellow, respectively. Amino acid residue sites that might be associated with neutralizing mAbs escape were in bold font. Sequences in gray were obtained from GenBank.

Rotavirus VP4 consists of two subunits, VP8* and VP5*, which contain four (8-1, 8-2, 8-3, and 8-4) and five (5-1, 5-2, 5-3, 5-4, and 5-5) neutralizing antigen epitope regions, respectively, involving a total of 37 neutralizing antigen epitope residues (Dormitzer et al., 2004). The VP4 neutralizing antigenic epitopes deduced from Chinese G1P[8] strains were mostly conserved, with only two amino acid variants found at aa site 89 for Fuzhou18-194 (2018) and Fuzhou18-262 (2018) strains. All the Chinese strains varied from the vaccine strains at seven sites, 150, 195, 113, 125, 131, 135, and 388, except that Fuzhou18-194 and Fuzhou18-262 showed an additional mutation site 89. Compared with Rotarix, the GS RVA strains and other Chinese G1P[8] of strains of earlier years had mutations at aa sites E150D, N195G, N113D, S125N, S131R, and N135D. Compared with RotaTeq, the Chinese strains varied at E150D, D195G, N113D, and L388I. The strains varied from Rotavin-M1 only at sites E150D, D195G, and S113D (**Supplementary Table 8**). By this, Chinese G1P[8] strain variations from Rotavin-M1 were lesser (four changes) compared to Rotarix and RotaTeq (seven and five changes). Conserved N-glycosylation sites were found at 32 aa and 589 aa in all Chinese G1P[8] and at 599 aa in most Chinese G1P[8] strains. However, all these sites were distant from neutralizing antibody escape sites.

**Figure 4:**
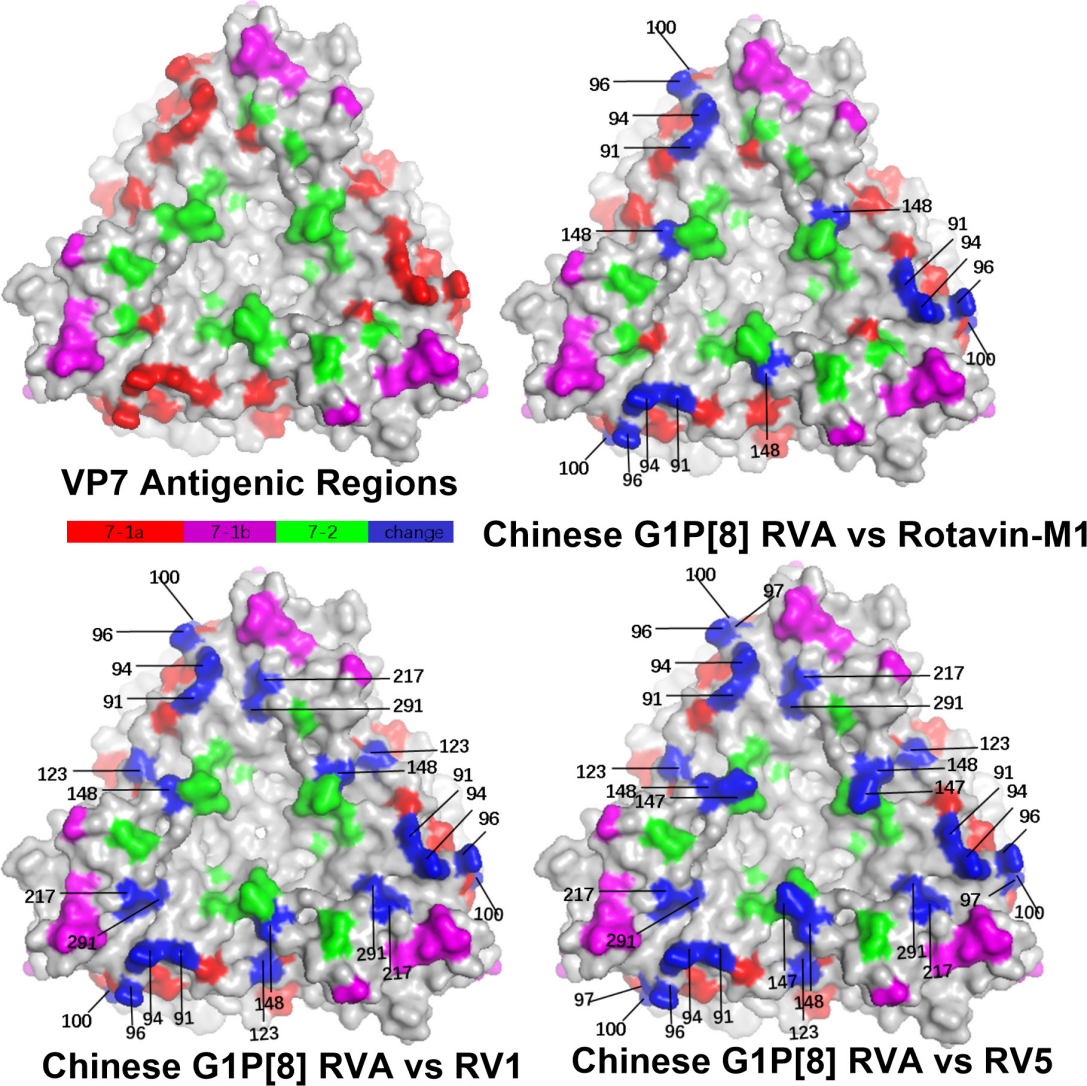
Presumed VP7 surface exposure antigen epitope amino acid variations between Chinese G1P[8] RVA and G1 vaccines Rotarix (RV1), RotaTeq (RV5), and Rotavin-M1 (PDB 3FMG) (Venkataram Prasad and Chiu, 1994). Surface-exposed antigenic epitopes on 7-1a, 7-1b, and 7-2 are represented by red, magenta, and green colors, respectively. Blue color shows antigen epitope variations between Chinese G1P[8] RVA and G1 vaccines.

**Figure 5:**
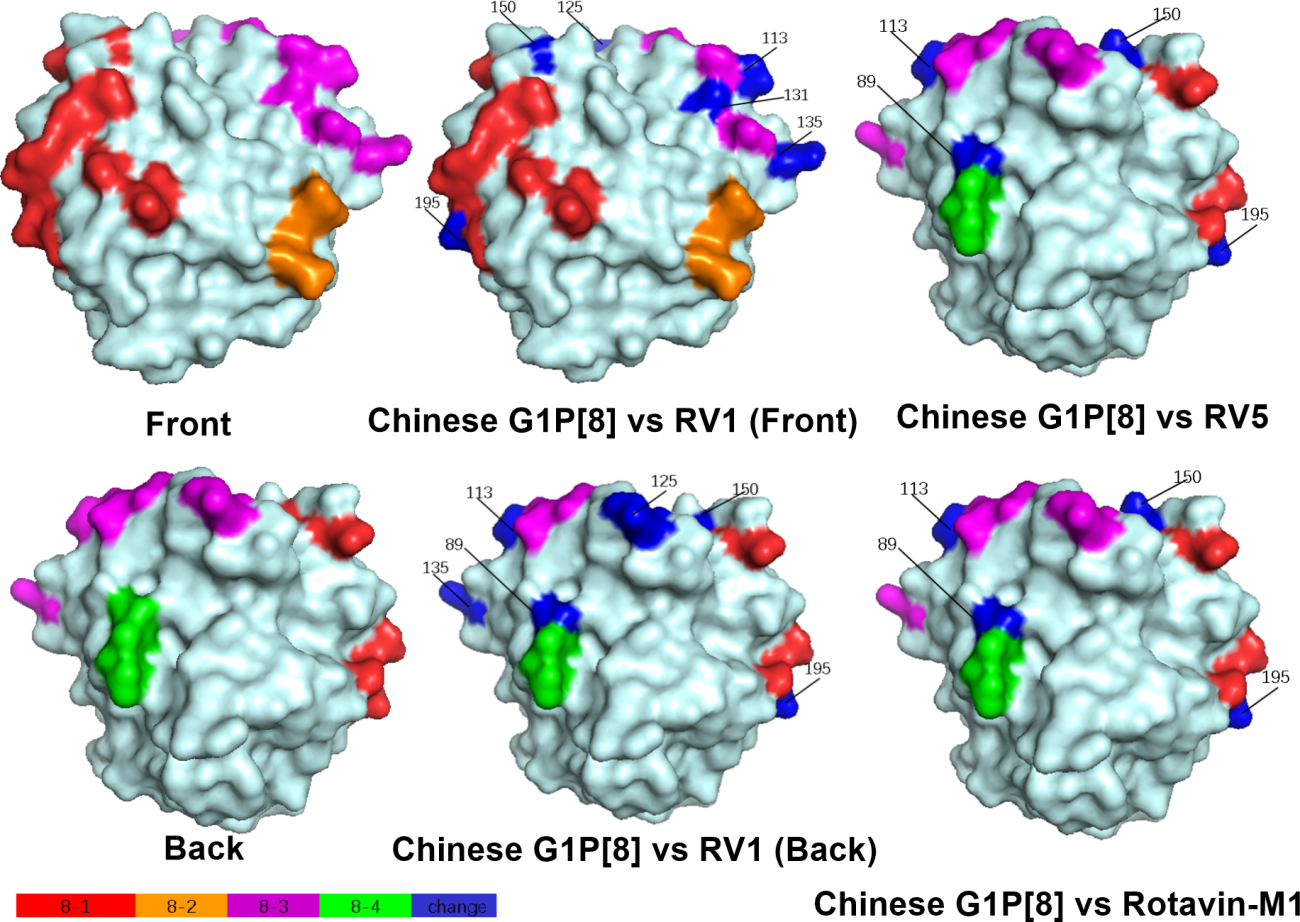
Presumed VP8* surface exposure antigen epitope amino acid variations between Chinese G1P[8] RVA and P[8] vaccines Rotarix (RV1), RotaTeq (RV5), and Rotavin-M1 (PDB 1KQR) (Dormitzer et al., 2002). Surface-exposed antigenic epitopes on 8-1, 8-2, 8-3, and 8-4 are represented by red, orange, magenta, and green colors, respectively. Blue color shows antigen epitope variations between Chinese G1P[8] RVA and P[8] vaccines.

Structural mapping of the VP7 protein trimer showed that the amino acid variants between the antigenic region of Chinese G1P[8] RVA and the three G1P[8] vaccines were widely distributed in the three-dimensional structure, exhibiting significant differences among them. The Chinese G1P[8] RVA had the lowest number of variants against Rotavin-M1, the next highest number of variants was with Rotarix, and the highest number of variants was with RotaTeq. These amino acid mutations were inconsistently located on the exposed surfaces of the protein in the epitope region, mainly on the 7-1a and 7-2 regions. 7-1b is conserved (**Figure 4**). The VP8* protein structure showed that the Chinese G1P[8] RVA had the most variants with Rotarix and the same number of variants with Rotavin-M1 and RotaTeq. These VP8* variant sites were inconsistently located in the antigenic epitope regions 8-1, 8-3, and 8-4, while 8-2 was conserved (**Figure 5**).

### Selection pressure analysis

After prediction by four selection pressure models, only one site 130 aa on the VP7 gene of G1P[8] RVA was under negative selection pressure (**Table 3**). The site was a neutralizing antigenic epitope of VP7 protein (Venkataram Prasad and Chiu, 1994). The VP4 gene received positive and negative selection pressure (**Tables 3**, **4**). One amino acid site 768 aa on the VP4 gene was under positive selection pressure. However, 13 amino acid sites, 147, 212, 218, 257, 326, 336, 337, 480, 525, 577, 592, 755, and 756, on the VP4 gene were under negative selection pressure. Of these, 147 aa was next to the VP4 protein neutralizing antigenic epitopes 146 and 148 (Dormitzer et al., 2004). The dN/dS of the coding regions of VP7 and VP4 genes was smaller than 1; i.e., the synonymous substitution rate was greater than the nonsynonymous substitution rate.

**Table 3:** Selection pressure analysis for the amino acid of G1P[8] RVA VP7 and VP4 genes in China.

Gene	Method	Positive	Negative	dN/dS*
VP7	MEME	0	0	0.151
	SLAC	0	1	0.156
	FEL	0	24	—
	FUBAR	0	19	—
	Integrated	0	1	
VP4	MEME	3	0	0.0974
	SLAC	1	13	0.104
	FEL	1	79	—
	FUBAR	3	104	—
	Integrated	1	13	

*Non-synonymous/synonymous rate ratio.

**Table 4:** Amino acid sites on VP4 under positive and negative selection pressure.

Methods	Positive sites	Negative sites
MEME	24, 768, 769	
SLAC	768	147, 212, 218, 257, 326, 336, 337, 480, 525, 577, 592, 755, and 756
FEL	768	79 sites such as 147, 212, 218, 257, 326, 336, 337, 480, 525, 577, 592, 755, and 756
FUBAR	24, 768, 769	104 sites such as 147, 212, 218, 257, 326, 336, 337, 480, 525, 577, 592, 755, and 756

### Bayesian evolutionary analysis of the VP7 and VP4 genes

**Figure 6:**
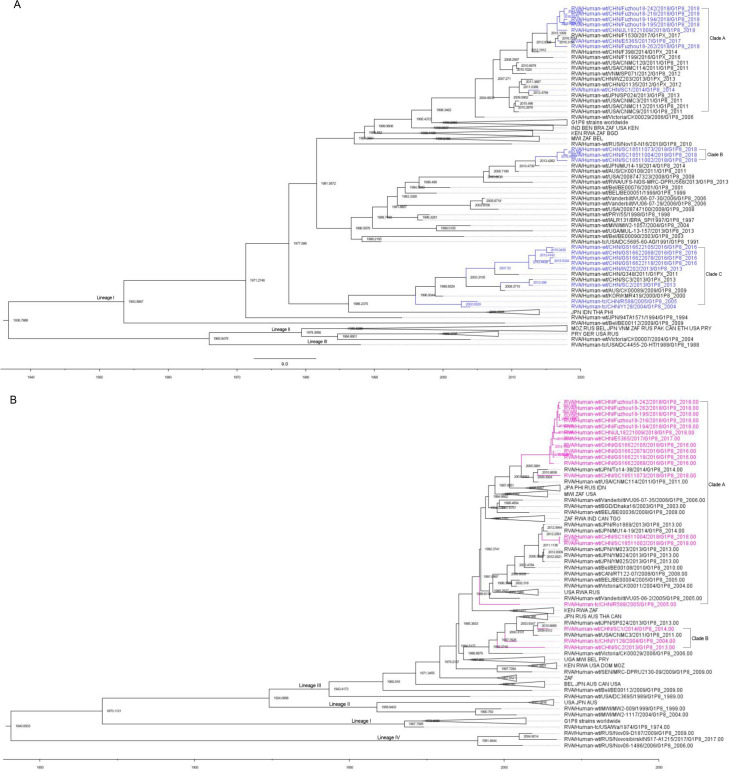
Maximum clade credibility (MCC) trees for the VP7 **(A)** and VP4 **(B)** genes of the G1P[8] rotavirus. The MCC trees were constructed using Bayesian Markov chain Monte Carlo analysis in BEAST v. 1.10.4. Reference strain sequences were obtained from GenBank (http://www.ncbi.nlm.nih.gov), and the tree included the names and sampling times of the strains. In the VP7 MCC tree **(A)**, Chinese G1P[8] strains were shown in blue, while in the VP4 MCC tree **(B)**, Chinese G1P[8] strains were shown in purple.

The results indicated that the mean evolutionary rate of VP7 of G1 was estimated to be 7.042 × 10^−4^ substitutions/site/year with a 95% high posterior density interval (95% HPD) of 5.7142 × 10^−4^ to 8.4576 × 10^−4^. The time of the most recent common ancestor (TMRCA) of the 167 G1 strains was calculated as 1,936.7888 (95% HPD interval: 1,910.07–1,957.10). The mean evolutionary rate of P[8] was estimated to be 6.97 × 10^−4^ substitutions/site/year (95% HPD interval: 5.23 × 10^−4^ to 8.78 × 10^−4^ substitutions/site/year). These 130 P[8] strains could be traced back to 1,840.6503 (95% HPD interval: 1,727.62–1,916.91). In terms of evolutionary dynamics, the analysis indicated that the VP7 gene of the G1P[8] RVA strains currently circulating in China was primarily distributed across three major evolutionary clades: Clade A and Clade B diverged around 1981, while Clade C diverged from them around 1977 (**Figure 6**). The VP4 gene of the G1P[8] RVA strains in China was mainly distributed in two major clades, which diverged around 1985 (**Figure 6**).

### Identity and phylogenetic tree

#### Identity and phylogenetic analysis of VP7

A total of 981 bp of GS RVA VP7 gene, encoding 326 amino acids, was analyzed. Identity analysis showed that VP7 of GS RVA in 2016–2018 was highly conserved with 99.6%–100% in nucleotide (nt) and 99.6%–100% in amino acid (aa) identities with each other. The nucleotide and amino acid identities of VP7 of GS RVA with that of other Chinese G1P[8] RVA from earlier years ranged from 92.5 to -99.8% and from 93.0% to 100%. The highest nt (99.8%) and aa (100%) identities were shared with WZ202 of 2013. The homology between the VP7 of GS RVA and the G1P[8] vaccines Rotarix (Rotarix-A41CB052A and Rotarix), RotaTeq (RotaTeq-WI79-9), and Rotavin-M1 (MW2-1026 and OP2-612) was 94.3%–94.4%, 91.0%–91.3%, and 97.2%–97.4% in nucleotide, and 94.1%–94.4%, 92.4%–92.7%, and 97.2%–97.9% in amino acid, respectively (**Supplementary Table 9**).

**Figure 7:**
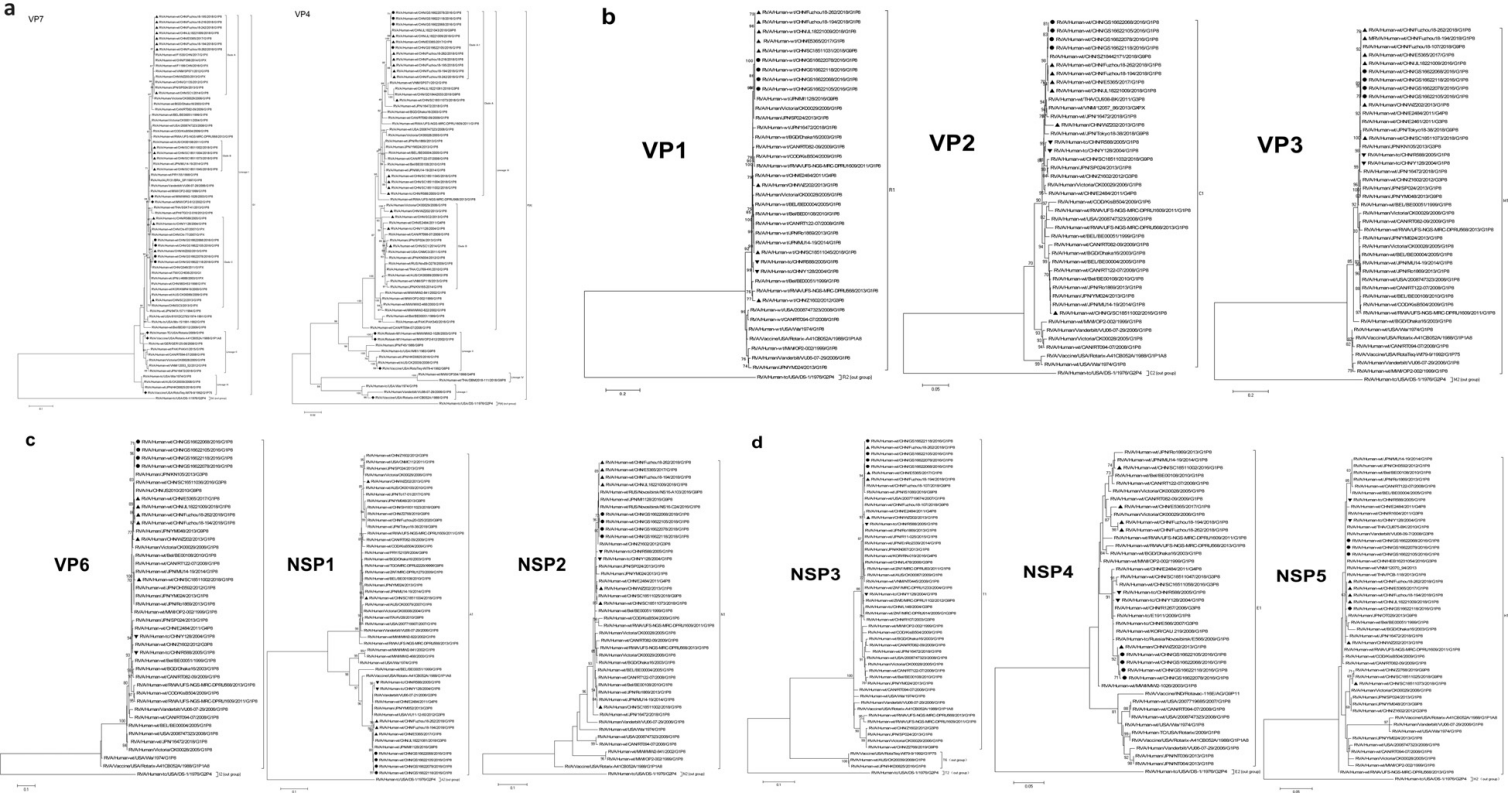
Maximum likelihood phylogenetic trees of G1P[8] RVA. Bootstrap values (1,000 replicates) are shown at branch nodes, and values <70% are hidden. Scale bars indicate distance. Black solid circular, GS G1P[8] RVA from China detected in this study. Black solid diamond, G1 genotype vaccines. Black solid triangle, other Chinese G1P[8] RVA strains before 2018. **(A)** VP7 phylogeny of G1 and VP4 phylogeny of P[8]. **(B)** VP1–VP3 phylogeny. **(C)** VP6, NSP1, and NSP2 phylogeny. **(D)** NSP3, NSP4, and NSP5 phylogeny.

Phylogenetic analysis showed that VP7 of GS RVA belonged to the G1-I lineage along with G1P[8] strains from earlier years of China and of other countries, although relatively distant from the latter. For example, the GS RVA formed Clade C with Chinese WZ202 and SC2 of 2013, R588 of 2005, and Y128 of 2004, indicating their close relationship. Also sharing the same Lineage I with the Chinese G1P[8] RVA, including GS RVA, was the Rotavin-M1, whereas the VP7 genes of vaccines Rotarix and RotaTeq are clustered in Lineage II and Lineage III, respectively (**Figure 7A**).

#### Identity and phylogenetic analysis of VP4

A total of 2,328 bp of GS RVA VP4 gene, encoding 776 amino acids, was analyzed. VP4 of GS RVA has 99%–100% nt and 100% aa identities with each other, which were highly conserved. Their identities with VP4 of other Chinese G1P[8] strains ranged from 93% to 99.9% in nucleotide and from 99.1% to 100.0% in amino acid, sharing the highest similarity values with E5365 and JL18221009 from 2017 and 2018 (99.8%–99.9% in nt and 100% in aa identities). The GS RVA also had high nucleotide and amino acid homology with Fuzhou strains like Fuzhou18-194 and Fuzhou18-262 from 2018 with values of 99.7%–99.8% (nt) and 99.7%–99.8% (aa), respectively. The VP4 identities between GS RVA and P[8] genotype vaccines Rotarix, RotaTeq, and Rotavin-M1 were 90.1%–90.2%, 92.3%–92.4%, and 93.0%–93.1% (nt), and 93.5%, 95.6%, and 96.4%–96.6% (aa), respectively (**Supplementary Table 9**).

By phylogenetic analysis, the VP4 of all Chinese G1P[8] RVA was located in the lineage P[8]-III. GS RVA was highly conserved and formed the separate Clade A-1 with E5365 from 2017, JL18221043, Fuzhou18-194, Fuzhou18-262, and other Fuzhou strains from 2018. The VP4 gene of Chinese G1P[8] RVA was distantly related to P[8] vaccines, with Rotavin-M1 and RotaTeq-WI79-4 located in Lineage II, and Rotarix-A41CB052A located in Lineage I (**Figure 7A**).

#### Identity and phylogenetic analysis of other genes

Except for NSP4, other genes of GS RVA showed similar patterns in homology and evolutionary characteristics to VP4 gene. By homology analysis, GS RVA strains were highly conserved with each other with 99.7%–100% nt and 99.3%–100% aa identities. The homology between GS RVA and other Chinese G1P[8] strains ranged 82.6%–100% nt and 80.2%–100% aa. Overall, GS RVA showed the highest homology with Fuzhou18-194 and Fuzhou18-262 from 2018 and E5365 from 2017, with 99.2%–100% nt and 98.3%–100% aa identities (**Supplementary Table 9**). In the phylogenetic trees of VP1–VP3, VP6, NSP1–NSP3, and NSP5, GS RVA and other Chinese G1P[8] strains were consistently located in the same lineage. The genetic relationship between GS RVA was usually closest, and they often formed small clades with JL18221043, Fuzhou18-194, and Fuzhou18-26 of 2018, and E5365 of 2017 (**Figures 7B–D**).

The NSP4 gene of GS RVA showed similar patterns in homology and evolutionary characteristics to VP7. GS RVA shared 99.6%–100% nt and 99.4%–100% aa sequence identities with each other. They had the highest homology with WZ202 of 2013 with 99.4%–99.8% nt and 99.4%–99.8% aa. However, they had low identities with Fuzhou18-194 and Fuzhou18-262 of 2018 and E5365 of 2017 with 94.4%–94.9% nt and 95.4%–96.5% aa (**Supplementary Table 9**). In the phylogenetic tree, NSP4 of GS RVA was on a small clade with WZ202 from 2013, indicating their close relationship, but distant from E5365, Fuzhou18-194, and Fuzhou18-262 relatively (**Figure 7D**).

## Discussion

This study aimed to understand the epidemiology and molecular and evolution characteristics of the G1P[8] genotype among all RVA in China on the eve of RotaTeq application, mainly in 2016–2018. The detection rate of G1P[8] in China was generally low at an average of 1.89% to 3.89% of all RVA infection. Peak detection rates of RVA in autumn–winter, different from the overall gastroenteritis cases, is a clear indication of seasonal specificity of the rotavirus infection. RVA seasonality with winter predominance has been observed in China and elsewhere (Colston et al., 2018; Satter et al., 2018; Tian et al., 2018; Ureña-Castro et al., 2019; Chinese Preventive Medicine Association, 2021). Interestingly, the detection of G1P[8] did not follow the autumn–winter preference but peaked including outside the typical RVA season. The consistent peak and low frequency throughout the year indicate the persistence of G1P[8] in China, suggesting that the genotype is unlikely to become extinct, potentially serving as a source for future high prevalence and outbreaks.

The composition ratio of G1P[8] fluctuated from 2016 to 2018 but generally showing decline, aligning with the decreasing trend observed by the Chinese CDC from 2010 to 2015 (data not shown). This decline in G1P[8] is not exclusive to China, as global trends show its reduction before the vaccine era since 2000, although the genotype remains prevalent in certain geographic locations such as Turkey, Tunisia, India, and Morocco (Hassine-Zaafrane et al., 2011; Durmaz et al., 2014; Mullick et al., 2014; Ezzine et al., 2021). The introduction of G1 vaccines (RotaTeq and Rotarix) led to a shift in genotype prevalence, notably reducing G1P[8] with the re-emergence of G2P[4] and the emergence of rare genotypes such as G12P[8] and the equine-like G3P[8] (Hungerford et al., 2019; Donato et al., 2022). Thus, although G1 vaccines have notably diminished the prevalence of G1P[8] through immunity against the G1 genotype, its decline predates the introduction of the vaccines. This brings to question if the vaccine introduction might further hasten the decline. The impact of the long-term use of the G10P[15] vaccine LLR since 2001 on the exceptionally low levels of G1P[8] in China before the RotaTeq era remains to be determined.

The emergence of drifted viruses, evading immunity from vaccination or natural infection, is anticipated. Convergent evolution favoring variants with epitope mutations crucial for immune evasion happened even with booster vaccination (Arista et al., 2006; Duerr et al., 2023). Given this, it is rational to utilize vaccines that closely match the target virus (Zeller et al., 2012). This study compares Chinese G1P[8] epitope characteristics with vaccines of homologous genotypes, Rotavin-M1, Rotarix, and RotaTeq, to assess their similarity to local strains. Results showed that, regardless of the neutralizing epitope (VP7 or VP4), the differences between Chinese G1P[8] and Rotavin-M1 were the least compared to Rotarix or RotaTeq, hinting at its potential superiority. Differences in VP4 or VP7 epitopes between epidemic strains and vaccines were shown not to reduce the protective effect vaccine (Kulkarni et al., 2014). Conserved N-glycosylation was also found at proximity to neutralizing epitopes or neutralizing antibody escape sites of the VP7 and VP4 of Chinese G1P[8], a phenomenon similarly observed in other countries (Jere et al., 2018; Sadiq and Bostan, 2020). These glycosylation sites bind sugar easily and may cause resistance of viruses to antibodies and reduce the protective effect of neutralizing antibody produced by the RotaTeq vaccine currently in use (Zeller et al., 2012; Gupta et al., 2021). Thus, the protective effect of RotaTeq, or the potential superiority of Rotavin-M1, against local strains needs to be verified through animal experiments and clinical trials. Additionally, negative selection on at least one neutralizing epitope on VP7 and at a site adjacent to the neutralizing epitope on VP4 is a strong reminder that both gene fragments are prone to purification selection pressure (Afrad et al., 2014). Considering this was observed even before the usage of RotaTeq in China, this prompts the question of how the vaccine will impact the selection pressure on G1P[8] VP7 and VP4 antigenicity.

The evolution behavior of G1P[8] in China before 2018 was evaluated, providing data support for future evolutionary analysis after the introduction of the RotaTeq vaccine in 2018. The mean evolution rate of G1 and P[8] in this study is 7.042 × 10^−4^ substitutions/site/year and 6.97 × 10^−4^ substitutions/site/year, similar to other studies (Tatsumi et al., 2006; Afrad et al., 2014; Fujii et al., 2019). Japan previously investigated the evolutionary dynamics of the emerging dominant genotype DS-1-like G1P[8], estimating an evolution rate of 7.26 × 10^−4^ nucleotide substitutions/site/year for the G1 strains. The analysis traced the nationwide G1 strains back to a common ancestor, possibly originating 2–6 years earlier (Fujii et al., 2019). Given the similarity in G1 evolutionary rates between this study and the Japanese, the year of the closest common ancestor identified for Japan’s G1 strains could also be applicable to China’s G1 strains. However, with RotaTeq introduced in Japan in 2011, the nationwide coverage of 39% potentially affected the evolution rate. A comparison with China’s G1P[8] evolutionary dynamics could provide clarity, particularly if conducted at least within similar number of years after the introduction of RotaTeq in 2018.

Next, the whole-genome sequences of G1P[8] from diarrhea stools of children in 2016 (GS RVA) were subjected to identity and evolutionary analyses along with Chinese G1P[8] strains from other times and reference sequences around the world. GS RVA had high homology and close evolutionary relationship with other Chinese G1P[8] strains, indicating the stability of their genome from before 2018. However, GS RVA G1P[8] gene fragments formed two clusters with other Chinese G1P[8]. VP7 and NSP4 of GS RVA shared the same clade with Chinese WZ202 (2013), indicating their close evolutionary relationship. The other gene fragments of GS RVA are more homologous and evolutionarily related to Chinese G1P[8] strain E5365 (2017) and Fuzhou18-194 (2018), among others. The separation suggests the potential for genetic recombination between VP7 and NSP4 fragments of the same genotype during local transmission from before 2018. This concept is not implausible, considering that intergenic recombination involving all RVA gene fragments is more common than previously understood, except for NSP3 and NSP5 gene fragments (Hoxie and Dennehy, 2020).

Rotarix and RotaTeq are widely used vaccines globally and have demonstrated their effectiveness in the Americas, Europe, and Australia (Coria de la, 2006; Vesikari et al., 2006). However, ongoing genetic evolution and mutations driven by selection pressure, as well as persistent infections, underline the need for continuous monitoring of the G1P[8] genotype (Carvalho-Costa et al., 2019b). This monitoring serves not only to assess the effectiveness of the RotaTeq vaccine but also to evaluate its impact on the evolution of Chinese G1P[8] strains. The epidemiological, molecular, and evolutionary characteristics of Chinese G1P[8], as investigated in this study conducted on the eve of the RotaTeq vaccine launch in the country, provide valuable data for predicting the future prevalence of G1P[8] in China and are essential for assessing vaccine efficacy.

## Conclusion

This study showed that the detection rate of G1P[8] in China from 2016 to 2018 was generally low and had significant seasonality. Neutralizing epitope analysis showed that Rotavin-M1 might have a better protective effect on G1P[8] prevalent in China than Rotarix and RotaTeq. Two conserved N-glycosylation sites on VP7 of Chinese G1P[8] might affect the protective effect of the vaccine. Evolution rate analysis and selection pressure analysis identified negative selection and indicated that G1P[8] had the possibility of rapidly evolving and adapting to the new environment introduced by vaccines, whereas positive selection specific to VP4 indicated the potential tendency to select for dominant traits. Identity and phylogeny analysis showed that Chinese G1P[8] from before 2018 were generally stable with possible genetic recombination among local strains. These findings are of great significance for predicting the prevalence of G1P[8] in China and evaluating vaccine efficacy.

## Data Availability

The datasets presented in this study can be found in online repositories. The names of the repository/repositories and accession number(s) can be found in the article/**Supplementary Material**.
