# Supplementary material for: Epidemiological, molecular, and evolutionary characteristics of G1P[8] rotavirus in China on the eve of RotaTeq application
Source: Front Cell Infect Microbiol. 2024 Dec 9;14:1453862. doi: 10.3389/fcimb.2024.1453862 (PMC11666228; doi:10.3389/fcimb.2024.1453862)
Supplement: Supplementary file 1 [file Table1.docx]

**Data**

All sequences used in this study are available in GenBank. All other relevant information is provided in this current manuscript. If required, the data presented in this work can be shared by e-mail.
