# Supplementary material for: Epidemiological, molecular, and evolutionary characteristics of G1P[8] rotavirus in China on the eve of RotaTeq application
Source: Front Cell Infect Microbiol. 2024 Dec 9;14:1453862. doi: 10.3389/fcimb.2024.1453862 (PMC11666228; doi:10.3389/fcimb.2024.1453862)
Supplement: Supplementary file 2 [file Table2.docx]

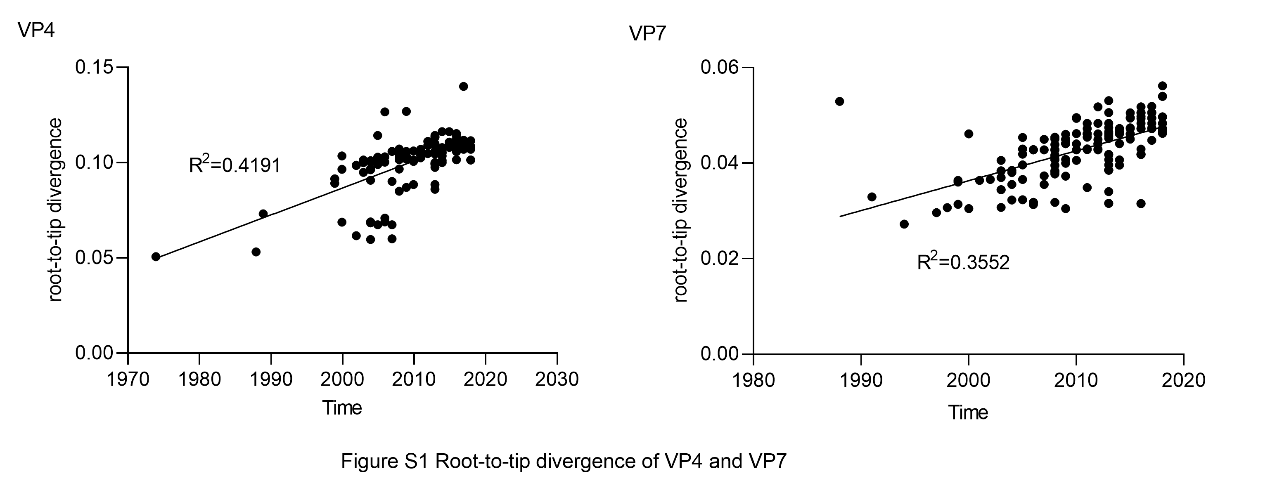


Root-to-tip regression analysis was performed using TempEst v1.5.3. The images confirmed that the dataset exhibited a temporal signal and all strains showed a linear rate of evolution.
