# Supplementary material for: Epidemiological, molecular, and evolutionary characteristics of G1P[8] rotavirus in China on the eve of RotaTeq application
Source: Front Cell Infect Microbiol. 2024 Dec 9;14:1453862. doi: 10.3389/fcimb.2024.1453862 (PMC11666228; doi:10.3389/fcimb.2024.1453862)
Supplement: Supplementary file 3 [file Table3.docx]

**Table S1 Model comparison for VP7 by marginal likelihood.**

| Trace | In P (data \| modle) | S.E. | Model 1 | Model 2 | Model 3 | Model 4 |
| --- | --- | --- | --- | --- | --- | --- |
| Model 1^a^ | -5416.519 | +/-0.688 | - | 12.918 | -10.214 | -18.377 |
| Model 2^b^ | -5429.437 | +/-0.164 | -12.918 | - | -23.132 | -31.295 |
| Model 3^c^ | -5406.305 | +/-0.156 | 10.214 | 23.132 | - | -8.163 |
| Model 4^d^ | -5398.142 | +/- | 18.377 | 31.295 | 8.163 | - |

^a^ Uncorrelated Relaxed Lognormal clock model, HKY+G substitution model, coalescent constant-size tree prior model.

^b^ strict clock model; HKY+G substitution model, coalescent constant-size tree prior model.

^c^ Uncorrelated Relaxed Lognormal clock model, GTR+G substitution model, coalescent constant-size tree prior model.

^d^ Uncorrelated Relaxed Lognormal clock model, GTR+G substitution model, Bayesian skyline coalescent tree prior model.

Models compared by marginal likelihood (S.E. estimated from bootstraps). Differences between log marginal likelihoods (specifically, log Bayes factors) are reported. Positive values indicate better relative model fit of the row’s model compared to the column’s model.
