# Supplementary material for: Epidemiological, molecular, and evolutionary characteristics of G1P[8] rotavirus in China on the eve of RotaTeq application
Source: Front Cell Infect Microbiol. 2024 Dec 9;14:1453862. doi: 10.3389/fcimb.2024.1453862 (PMC11666228; doi:10.3389/fcimb.2024.1453862)
Supplement: Supplementary file 7 [file Table7.docx]

| **Table S3 Chi-Square tests for RVA detection rates by month** | | | |
| --- | --- | --- | --- |
|  | Value | df | Asymp. Sig. (2-sided) |
| Pearson Chi-Square | 2.120E3^a^ | 11 | .000 |
| Likelihood Ratio | 2.235E3 | 11 | .000 |
| Linear-by-Linear Association | 311.757 | 1 | .000 |
| N of Valid Cases | 15374 |  |  |
| a. 0 cells (.0%) have expected count less than 5. The minimum expected count is 270.75. | | | |

| **Table S4 Chi-Square tests for G1P[8] genotype from all acute gastroenteritis cases by month** | | | |
| --- | --- | --- | --- |
|  | Value | df | Asymp. Sig. (2-sided) |
| Pearson Chi-Square | 99.627^a^ | 11 | .000 |
| Likelihood Ratio | 87.479 | 11 | .000 |
| Linear-by-Linear Association | 37.468 | 1 | .000 |
| N of Valid Cases | 15374 |  |  |
| a. 0 cells (.0%) have expected count less than 5. The minimum expected count is 7.60. | | | |

| **Table S5 Chi-Square Tests for G1P[8] composition ratios by month** | | | |
| --- | --- | --- | --- |
|  | Value | df | Asymp. Sig. (2-sided) |
| Pearson Chi-Square | 30.809^a^ | 11 | .001 |
| Likelihood Ratio | 32.046 | 11 | .001 |
| Linear-by-Linear Association | 9.546 | 1 | .002 |
| N of Valid Cases | 4309 |  |  |
|  |  |  |  |
| a. 4 cells (16.7%) have expected count less than 5. The minimum expected count is 2.72. | | | |
