# Supplementary material for: Epidemiological, molecular, and evolutionary characteristics of G1P[8] rotavirus in China on the eve of RotaTeq application
Source: Front Cell Infect Microbiol. 2024 Dec 9;14:1453862. doi: 10.3389/fcimb.2024.1453862 (PMC11666228; doi:10.3389/fcimb.2024.1453862)
Supplement: Supplementary file 8 [file Table8.docx]

**Supplementary Table 1 Presumed VP4 neutralizing antigen epitope differences between Chinese G1P[8] RVA and G1P[8] vaccines.**

| Strains | 8-1 | | | | | | | | | | | 8-2 | | 8-3 | | | | | | | | | 8-4 | | | 5-1 | | | | | | | | 5-2 | 5-3 | 5-4 | 5-5 |
| --- | --- | --- | --- | --- | --- | --- | --- | --- | --- | --- | --- | --- | --- | --- | --- | --- | --- | --- | --- | --- | --- | --- | --- | --- | --- | --- | --- | --- | --- | --- | --- | --- | --- | --- | --- | --- | --- |
|  | **100** | **146** | **148** | **150** | **188** | **190** | 192 | 193 | **194** | 195 | 196 | **180** | **183** | 113 | **114** | 115 | **116** | 125 | 131 | **132** | **133** | **135** | **87** | **88** | **89** | **384** | **386** | **388** | **393** | **394** | **398** | **440** | **441** | **434** | **459** | **429** | **306** |
| RV1/G1P[8] (Rotarix) | D | S | Q | E | S | T | N | L | N | N | I | T | A | N | P | V | D | **S** | **S** | N | D | **N** | N | T | N | Y | F | I | W | P | G | R | T | P | E | L | R |
| RV5/P[8] (RotaTeq) | D | S | Q | E | S | T | N | L | N | D | I | T | A | N | P | V | D | N | R | N | D | D | N | T | N | Y | F | L | W | P | G | R | T | P | E | L | R |
| Rotavin-M1/P[8] | D | S | Q | E | S | T | N | L | N | D | I | T | A | S | P | V | D | N | R | N | D | D | N | T | N | Y | F | I | W | P | G | R | T | P | E | L | R |
| GS16622068/2016 | D | S | Q | D | S | T | N | L | N | G | I | T | A | D | P | V | D | N | R | N | D | D | N | T | N | Y | F | I | W | P | G | R | T | P | E | L | R |
| GS16622078/2016 | D | S | Q | D | S | T | N | L | N | G | I | T | A | D | P | V | D | N | R | N | D | D | N | T | N | Y | F | I | W | P | G | R | T | P | E | L | R |
| GS16622105 | D | S | Q | D | S | T | N | L | N | G | I | T | A | D | P | V | D | N | R | N | D | D | N | T | N | Y | F | I | W | P | G | R | T | P | E | L | R |
| GS16622118 | D | S | Q | D | S | T | N | L | N | G | I | T | A | D | P | V | D | N | R | N | D | D | N | T | N | Y | F | I | W | P | G | R | T | P | E | L | R |
| Fuzhou 18-194/2018 | D | S | Q | D | S | T | N | L | N | G | I | T | A | D | P | V | D | N | R | N | D | D | N | T | **S** | Y | F | I | W | P | G | R | T | P | E | L | R |
| Fuzhou18-262/2018 | D | S | Q | D | S | T | N | L | N | G | I | T | A | D | P | V | D | N | R | N | D | D | N | T | **S** | Y | F | I | W | P | G | R | T | P | E | L | R |
| Fuzhou18-195/2018 | D | S | Q | D | S | T | N | L | N | G | I | T | A | D | P | V | D | N | R | N | D | D | N | T | **N** | Y | F | I | W | P | G | R | T | P | E | L | R |
| Fuzhou18-216/2018 | D | S | Q | D | S | T | N | L | N | G | I | T | A | D | P | V | D | N | R | N | D | D | N | T | **N** | Y | F | I | W | P | G | R | T | P | E | L | R |
| Fuzhou18-242/2018 | D | S | Q | D | S | T | N | L | N | G | I | T | A | D | P | V | D | N | R | N | D | D | N | T | **S** | Y | F | I | W | P | G | R | T | P | E | L | R |
| SC18511002/2018 | D | S | Q | D | S | T | N | L | N | G | I | T | A | D | P | V | D | N | R | N | D | D | N | T | **S** | Y | F | I | W | P | G | R | T | P | E | L | R |
| SC18511004/2018 | D | S | Q | D | S | T | N | L | N | G | I | T | A | D | P | V | D | N | R | N | D | D | N | T | N | Y | F | I | W | P | G | R | T | P | E | L | R |
| SC18511045/2018 | D | S | Q | D | S | T | N | L | N | G | I | T | A | D | P | V | D | N | R | N | D | D | N | T | N | Y | F | I | W | P | G | R | T | P | E | L | R |
| SC18511073/2018 | D | S | Q | D | S | T | N | L | N | G | I | T | A | D | P | V | D | N | R | N | D | D | N | T | N | Y | F | I | W | P | G | R | T | P | E | L | R |
| E5365/2017 | D | S | Q | D | S | T | N | L | N | G | I | T | A | D | P | V | D | N | R | N | D | D | N | T | N | Y | F | I | W | P | G | R | T | P | E | L | R |
| SC1/2014 | D | S | Q | D | S | T | N | L | N | G | I | T | A | D | P | V | D | N | R | N | D | D | N | T | N | Y | F | I | W | P | G | R | T | P | E | L | R |
| WZ202/2013 | D | S | Q | D | S | T | N | L | N | G | I | T | A | D | P | V | D | N | R | N | D | D | N | T | N | Y | F | I | W | P | G | R | T | P | E | L | R |
| SC2/2013 | D | S | Q | D | S | T | N | L | N | G | I | T | A | D | P | V | D | N | R | N | D | D | N | T | N | Y | F | I | W | P | G | R | T | P | E | L | R |
| R588/2005 | D | S | Q | D | S | T | N | L | N | G | I | T | A | D | P | V | D | N | R | N | D | D | N | T | N | Y | F | I | W | P | G | R | T | P | E | L | R |
| Y128/2004 | D | S | Q | D | S | T | N | L | N | G | I | T | A | D | P | V | D | N | R | N | D | D | N | T | N | Y | F | I | W | P | G | R | T | P | E | L | R |

Amino acid residues highlighted in blue were those different from all three vaccines Rotarix (RV1), RotaTeq (RV5) and Rotavin-M1. Amino acid residues only different from Rotarix or RotaTeq was marked with purple or red, respectively. Amino acid residue sites associated with neutralizing mAbs escape were in bold font. Sequences in grey were obtained from the GenBank.
